# Next Generation Methods for Single-Molecule Force Spectroscopy on Polyproteins and Receptor-Ligand Complexes

**DOI:** 10.3389/fmolb.2020.00085

**Published:** 2020-05-19

**Authors:** Byeongseon Yang, Zhaowei Liu, Haipei Liu, Michael A. Nash

**Affiliations:** ^1^Department of Chemistry, University of Basel, Basel, Switzerland; ^2^Department of Biosystems Science and Engineering, ETH Zürich, Basel, Switzerland

**Keywords:** single-molecule biophysics, molecular engineering, AFM, protein stability and folding, molecular biomechanics

## Abstract

Single-molecule force spectroscopy with the atomic force microscope provides molecular level insights into protein function, allowing researchers to reconstruct energy landscapes and understand functional mechanisms in biology. With steadily advancing methods, this technique has greatly accelerated our understanding of force transduction, mechanical deformation, and mechanostability within single- and multi-domain polyproteins, and receptor-ligand complexes. In this focused review, we summarize the state of the art in terms of methodology and highlight recent methodological improvements for AFM-SMFS experiments, including developments in surface chemistry, considerations for protein engineering, as well as theory and algorithms for data analysis. We hope that by condensing and disseminating these methods, they can assist the community in improving data yield, reliability, and throughput and thereby enhance the information that researchers can extract from such experiments. These leading edge methods for AFM-SMFS will serve as a groundwork for researchers cognizant of its current limitations who seek to improve the technique in the future for in-depth studies of molecular biomechanics.

## Introduction

Single-molecule force spectroscopy (SMFS) is a well-established method that directly probes structural changes of macromolecules under the influence of mechanical force. Since mechanical forces are ubiquitous in biology, insights gleaned from SMFS experiments shed light onto fundamentally important molecular mechanisms by which biological systems are able to sense, transduce and generate mechanical forces *in vivo*. Several native biological systems where mechanical forces play a significant role have been investigated with SMFS, including examples from muscle (Rief et al., 1997; Puchner et al., 2008a; Rivas-Pardo et al., 2016; Eckels et al., 2019), hearing (Lee et al., 2006; Bartsch et al., 2019; Hazra et al., 2019; Mulhall et al., 2019; Oroz et al., 2019), blood coagulation (Kim et al., 2010; Zhmurov et al., 2011; Müller et al., 2016), cell adhesion (Zhang et al., 2002; Marshall et al., 2003; Evans and Calderwood, 2007), the extracellular matrix (Oberhauser et al., 1998, 2002), protein folding at the ribosomal exit tunnel (Goldman et al., 2015; Guinn et al., 2018; Samelson et al., 2018), protein unfolding and proteolysis by the proteasome (Aubin-Tam et al., 2011; Cordova et al., 2014), and DNA/RNA molecular motors (Gelles and Landick, 1998; Neuman et al., 2003; Bornschlögl et al., 2009; Milic et al., 2014) to name but a few.

Single-molecule force spectroscopy can also be used to probe non-mechanical proteins and provide insights into their functionality as well. The free energy landscape (Woodside and Block, 2014), which is a theoretical space of high dimensionality on which a protein molecule diffuses and samples different conformations, is a general concept which applies to all proteins. Researchers have come to appreciate that conceptually, the application of mechanical force tilts this underlying energy landscape and forces the molecule under investigation to sample conformations along a specific reaction coordinate in an accelerated manner. This allows researchers to observe conformational changes and reactions that might otherwise be too slow to observe experimentally, and to quantify discrete states of a molecule that may be transient in the absence of force but biologically relevant nonetheless. These rare states can be converted into highly populated states when the energy landscape is biased by force. Both mechanical proteins and non-mechanical proteins are therefore valid targets for study by SMFS.

One area where SMFS on non-mechanical proteins could play an important role in the future is in the development of therapeutic proteins in the biopharma industry. Biophysical stability of therapeutic antibodies and other binding scaffold proteins is known to be predictive of their developability (Jain et al., 2017; Golinski et al., 2019). This means that even if an antibody binds its target and achieves its biological goal of, for example, influencing a signaling pathway, that alone does not make the molecule a viable drug. Therapeutic molecules must be colloidally and biophysically stable (Rabia et al., 2018; Xu et al., 2019) in order not to denature or aggregate under exposure to shear stress and other biophysical challenges encountered during manufacture, storage, shipping, and administration. The biopharmaceutical industry is therefore interested in methods that can accelerate the ability to screen candidate molecules at an early stage and determine their biophysical stability, and SMFS can contribute in that effort.

One criticism that is sometimes launched at SMFS is that the application of force to study non-mechanical protein folding reactions is somehow unnatural if the protein under investigation is not involved in mechanical force transduction natively. The force spectroscopy community would counter this argument by noting firstly that, in order to study protein folding we need to perturb the native state somehow and that, in fact mechanical force is probably a more natural denaturing stimulus than the other commonly accepted approaches such using high temperatures or denaturing salts or solvents to unfold proteins. In reality, mechanical force is very physiological.

Despite the high-potential for SMFS to elucidate mechanisms in biology and contribute to the development of biophysically stable therapeutics, in 2020 the technique remains a niche that has not been widely adopted by the greater molecular biosciences community. There are at least three reasons for this. The first is the specialized equipment required to perform such measurements. Currently, the range of experimental apparatus commonly used for performing SMFS experiments include optical tweezers (Neuman and Nagy, 2008), magnetic tweezers (Gosse and Croquette, 2002), centrifugal force microscopy (Yang et al., 2016), acoustic force spectroscopy (Sitters et al., 2015), biomembrane force probe (Merkel et al., 1999), and the instrument that is the focus for the current review, the atomic force microscope (Binnig et al., 1986). These instruments were uniformly born from the field of physics, and many still require researchers to build their own customized setups which slows adoption of these techniques. Secondly, there are severe challenges associated with performing SMFS inside of cells (Dufrêne et al., 2011), which for many researchers is a non-starter. Finally, single-molecule measurements are very sensitive to artifacts and care must be taken when choosing which trajectories represent valid single-molecule interaction traces. This fact could lead some researchers to believe that the technique is unreliable.

The purpose of this focused review is to highlight recent advances in AFM-based SMFS methodology that address the existing limitations and improve aspects such as sample throughput, sensitivity, reliability, and general robustness of the measurement. There are several recent reviews on related topics that overlap with the current review (Chen et al., 2015; Hughes and Dougan, 2016; Schönfelder et al., 2016a, 2018; Johnson and Thomas, 2018; Li and Zheng, 2018; Nathwani et al., 2018), and we regret that we were not able to include all the relevant work. We have organized the review into three sections. In the first section, we describe the various measurement configurations that are available in AFM-SMFS. We describe various formats in which a molecule (usually a protein) of interest can be presented and probed in an AFM-SMFS experiment. The second section then addresses theoretical considerations for analyzing AFM-SMFS datasets, as well as algorithms to extract maximal information from hard earned data traces. In the third section, we describe bioconjugation strategies for immobilizing proteins with site-specific attachment to surfaces and cantilevers for AFM-SMFS and describe recent approaches to protein-ligation which can facilitate novel measurement formats. This focused summary of methods should be helpful in planning and executing AFM-SMFS experiments in order to bring the technique to a wider range of researchers in the future.

## Measurement Configurations for Afm-Smfs

**FIGURE 1:**
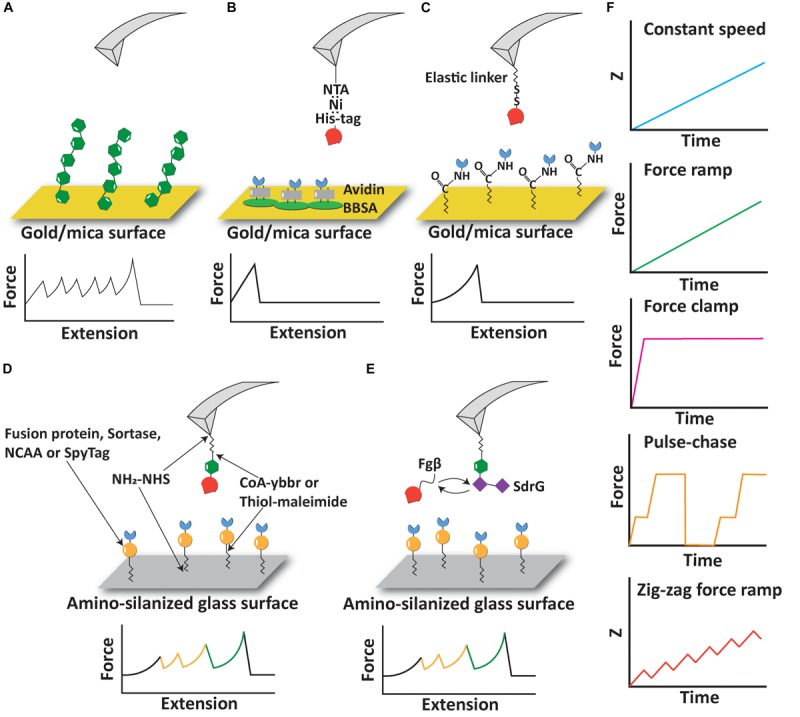
Experimental configurations and pulling protocols of AFM-SMFS. **(A)** AFM measurement based on non-specific adsorption of proteins. **(B)** Immobilization of proteins using non-covalent interactions including His:Ni and biotin:avidin. **(C)** Immobilization of proteins using elastic linkers and covalent bonds. **(D)** Covalent immobilization of proteins of interest and fingerprint domains using a variety of reactions and peptide tags. **(E)** A free diffusion system allows continuous exchange of ligand molecules on the cantilever. **(F)** Different pulling protocols used in AFM-SMFS.

The term “polyprotein” in this context refers to a protein containing multiple subdomains that mechanically fold/unfold independently of one another. One of the earliest configurations for AFM-SMFS on proteins relied on non-specific adsorption of polyproteins onto adsorptive surfaces, most often gold or mica (Rief et al., 1997, 1999; Oberhauser et al., 1998; Oesterhelt et al., 2000). The AFM cantilever tip is brought into contact with a surface sparsely decorated with adsorbed polyproteins, and with a low probability, a single molecule non-specifically adsorbs onto the AFM cantilever tip forming a tether between the cantilever and the surface, as shown in Figure 1A. This strategy controls the loading geometry on individual domains between their N- and C-terminus. Although the pickup point within the protein is not controlled, there are many copies of the domain within the polyprotein so the method ensures that at least several events in the resulting data traces represent controlled loading of the domain between the N- and C- termini. Several successful examples of non-specific pickup being used to quantify folding/unfolding rates and folding intermediate states in polyproteins have been reported over the years (Schwaiger et al., 2005; Bornschloegl and Rief, 2011), and the technique remains in use until today (Scholl and Marszalek, 2018).

Despite the success of AFM-SMFS on non-specifically adsorbed polyproteins, there remain several limitations of the technique. One aspect that should be considered in polyprotein stretching experiments in a constant speed scenario is the so-called “N-effect” (Zinober et al., 2009; King et al., 2010; Cao and Li, 2011; Tych et al., 2015) which leads to an underestimation of the unfolding forces for domains that unfold early in the sequence and can skew the energy landscape parameters. Since there are more domains available to unfold in a given time step at the beginning of a polyprotein stretching curve, lower unfolding forces are observed for domains early in the trace. The sawtooth-like peaks then tend to increase in magnitude as fewer and fewer domains remain folded at later stages of the trace. Counteracting this is the elasticity of the linker, which originates from an increase in the length and compliance of the unfolded linker region as the domains unfold in series and additional contour length is released from the folded structures. When probed in force clamp or force ramp mode (see below), the N-effect does not play a role (Cao and Li, 2011). When non-specific protein adsorption is used, the number of domains probed is not strictly controlled. A second limitation of the non-specific polyprotein approach is that the yield of useable single-molecule interaction curves is very low, sometimes well below 1%. This is because non-specific pickup of polyproteins is unpredictable, prone to spurious signals, and in many ways unreliable. More modern bioconjugate techniques have been developed to improve the pickup probability of sparsely populated molecules on the surface to address this limitation (see below). Finally, there remains the limitation that non-specific pickup procedures are not useful for probing receptor-ligand interactions because molecules that are picked up will clog the AFM tip and complicate data analysis.

Receptor-ligand interactions are a major class of protein-protein interactions that pose interesting objects of study for the AFM-SFMS community. Receptor-ligand interactions probed by AFM-SFMS have been reported both as the object of study as well as a tool for improving experimental yields in AFM-SMFS experiments using an approach referred to as “molecular handles” (Otten et al., 2014). Early in the development of receptor-ligand handles for AFM-SMFS, several affinity tags commonly found in protein biochemistry labs were used to pick up molecules with the AFM. For example, high-affinity non-covalent interactions including Ni:His-tag and Biotin:Avidin have both been used as immobilization tags onto surfaces or as the object of study in AFM-SMFS (Fritz et al., 1998; Berquand et al., 2005; Dupres et al., 2005; Li et al., 2019), as shown in Figure 1B. Many different classes of receptor-ligands have been probed by AFM (Milles et al., 2017; Liu et al., 2019), including antibody-antigen interactions (Dammer et al., 1996; Hinterdorfer et al., 1996, 1998; Allen et al., 1997; Ros et al., 1998; Schwesinger et al., 2000; Kienberger et al., 2005; Sulchek et al., 2005, 2006; Neuert et al., 2006; Odorico et al., 2007; Morfill et al., 2008; Roy et al., 2010; Bizzarri and Cannistraro, 2014; Klamecka et al., 2015; Tsai et al., 2016; Liu et al., 2019, 2020), avidin systems (see below), bacterial adhesion systems (Herman et al., 2014; Schoeler et al., 2014; Jobst et al., 2015; Nash et al., 2016; Alonso-Caballero et al., 2018; Herman-Bausier et al., 2018; Milles et al., 2018; Viela et al., 2019), and others (Sletmoen et al., 2004; Farrance et al., 2013).

The biotin-(strept)avidin interaction has a long history in the AFM-SMFS community (Florin et al., 1994, 1995; Lee et al., 1994; Moy et al., 1994; Grubmüller et al., 1996; Wong et al., 1999; Yuan et al., 2000; Pincet and Husson, 2005; de Odrowaz Piramowicz et al., 2006; Rico and Moy, 2007; Erdmann et al., 2008; Guo et al., 2008; Zhang et al., 2009; Chivers et al., 2010; Taninaka et al., 2010a, b; Teulon et al., 2011; Han et al., 2012; Rico et al., 2015, 2019; Baumann et al., 2016; Erlich et al., 2017; Sedlak et al., 2017, 2019, 2020; Bauer et al., 2018), and a thorough review of this controversy is beyond the scope of the review here. Much of the irreproducibility of biotin-streptavidin rupture force measurements by AFM-SMFS can be attributed to random lysine-based immobilization of the protein, as well as the fact that the streptavidin tetramer can disassemble during stretching. This leads to many different unbinding reaction pathways that need to be carefully disentangled to provide quantitative results. His-tag systems are often used to immobilize a receptor protein onto an AFM cantilever tip or sample surface modified with Ni-NTA, which provides an easy way to control the geometric pulling configurations on the receptor complex by placing a histidine tag at a specific position (typically N- or C-terminus). Site-specific biotinylation tags are furthermore available using the biotin-ligase BirA (Beckett et al., 1999; de Boer et al., 2003; Chen et al., 2005), and site-specific biotinylation of recombinant proteins is a valuable method recently reported for magnetic tweezers-based SMFS measurements (Renn et al., 2019). In addition to the biotin ligase acceptor sequence, the Streptag peptide sequence is being commonly used for AFM-SMFS with success (Baumann et al., 2016; Erlich et al., 2017). One limitation of the aforementioned non-covalent interactions as molecular handles for AFM-SMFS is the relatively low forces required to rupture these complexes. Both biotin/avidin and Ni-NTA/His-tag pairs break at around ∼100–200 pN depending on the loading rate (Florin et al., 1994; Kienberger et al., 2000). Therefore, depending on the strength of the domain(s) involved, these receptor-ligand may not be suitable as handles to stretch and unfold mechanostable domains fused with them.

Another commonly used measurement configuration involves covalent bond formation between the protein of interest (POI) and the surface. Since the rupture force of a covalent bond is >2 nN (Grandbois et al., 1999), covalent linkage to the surface establishes a link that is significantly more stable than typical receptor-ligand interactions or domain unfolding forces. Covalent linkage of proteins to surfaces/AFM tips is therefore a suitable setup for measuring mechanostable protein interactions and domain unfolding. Such an approach is also valuable when combined with the approaches mentioned, particular specific receptor-ligands as pulling handles. As shown in Figure 1C, disulfide bonds and EDC/NHS coupling reactions were used to covalently link cysteine or lysine residues to the surface via a polyethylene glycol (PEG) linker (Hinterdorfer et al., 1996; Berquand et al., 2005). Disulfide bonds have also been used to measure the unfolding force of single protein domains under different pulling geometries (Dietz et al., 2006). However, strictly defining the pulling geometry in this case may be hampered by native cysteines or the multiplicity of lysines present on the POI.

## Polyproteins Assembled by Receptor-Ligand Complexes

A drawback of the experimental configurations reported above for AFM-SMFS on receptor-ligand interactions is that valid single-molecule interactions are difficult to discriminate from non-specific interactions or multiple interactions occurring in parallel (Guo et al., 2008; Johnson and Thomas, 2018). Although the elastic linker attaching the protein to the surface helps to exclude short range non-specific adhesion (Tong et al., 2013), it is not sufficient to eliminate all background signals. To solve this problem, experimenters have identified a variety of protein domains which have characteristic unfolding patterns, well-defined contour lengths and unfolding forces that can serve as internal control modules to validate single-molecule interactions. These protein domains are known as “fingerprint domains” and have been used to screen for single receptor-ligand complex unbinding events from large datasets. We note that the fingerprint domains used for receptor-ligand SMFS should be chosen so that they unfold at a much lower range of forces than the unbinding event of the receptor-ligand under study in order to avoid the fingerprint biasing effect (Schoeler et al., 2016).

A typical AFM experimental setup to measure protein-ligand interactions with fingerprint domains is shown in Figure 1D. A polyprotein consisting of a fingerprint domain and the protein/ligand of interest is covalently immobilized on the AFM tip or the surface through an elastic linker, most often a poly(ethylene glycol) (PEG) linker (Zimmermann et al., 2010) or more recently an elastin-like polypeptide (ELP) (Ott et al., 2017). The POI can be expressed as a fusion protein with the fingerprint domain or covalently attached to the fingerprint domain and elastic linker using sortase or ybbr tags (Durner et al., 2017; Ott et al., 2017; Liu et al., 2018). A broad range of receptor-ligand interactions including cohesin-dockerin (Schoeler et al., 2014, 2015; Milles et al., 2017; Bernardi et al., 2019), antibody-antigen (Liu et al., 2019) and bacterial adhesin-host interactions (Milles et al., 2018) have been studied with the help of fingerprint domains.

In the aforementioned experimental setup, the protein-ligand interaction can be lost due to irreversible unfolding of the protein molecule immobilized on the tip. In order to solve this problem, an exchangeable receptor-ligand pair, SdrG:Fgβ, was added between the receptor and ligand, as shown in Figure 1E. Two features of the SdrG:Fgβ complex are crucial to this experimental configuration: (1) the SdrG:Fgβ complex is able to withstand a force as high as 2 nN (Milles et al., 2018), which is in the same regime as a covalent bond and significantly larger than other receptor-ligand interactions. Therefore the receptor-ligand complex would always rupture without breaking the SdrG:Fgβ interaction; and (2) the affinity between SdrG and Fgβ is moderate (300–400 nM) (Ponnuraj et al., 2003). Therefore, the receptor/ligand molecule attached to the tip is frequently exchanged based on the natural off-rate at equilibrium of this complex. A freely diffusing molecule can then re-bind the SdrG molecule on the tip and prevent the loss of interaction due to tip clogging or protein unfolding. This experimental setup has been used to characterize the mechanical properties of monovalent and tetravalent streptavidin:biotin complex (Sedlak et al., 2019, 2020). A limitation of this method is that the N terminus of the Fgβ peptide has to be exposed to interact with SdrG, which restricts the geometry and necessitates that the Fgβ peptide is located at the N terminus of the freely diffusing molecule, and that the receptor-of-interest is situated at the C-terminal of the freely diffusing molecule. An overview of selected fingerprint domains is listed in Table 1. While some of these fingerprint domains have been probed as standard polyproteins, others were used in polyproteins assembled through mechanostable receptor-ligand interactions. Due to differences in cantilever stiffness and data analysis procedures among the various studies, values in the table should be considered approximations.

**TABLE 1:** Overview of selected fingerprint domains.

**Fingerprint domain**	**Approximate unfolding force [pN]**	**Pulling speed [nm/s]**	**Approximate contour length increment [nm]**	**References**
^10^FNIII	90	400	32	Li et al., 2005
*A. cellulolyticus* ScaA Cohesins	Coh1: 139 Coh2: 402 Coh3: 346 Coh4: 578 Coh5: 587 Coh6: 461 Coh7: 523	1600	45	Verdorfer et al., 2017
ARNT PAS-B	33	400	39	Gao et al., 2012
C3 cardiac myosin binding protein	90	40 pN⋅s^–1^ [force ramp]	43	Karsai et al., 2011; Pimenta-Lopes et al., 2019
CD4D1 CD4D2	130 100	400	8.2 13.3	Perez-Jimenez et al., 2014
Cellulose binding module (CBM)	150	200–6400	58	Schoeler et al., 2014; Liu et al., 2018
Csp	80	400	24	Schönfelder et al., 2016b
DHFR	82	400	67	Ainavarapu et al., 2005; Junker et al., 2005
ddFLN4	2 unfolding steps, step 1: 56, step 2: 48	250–350	14 (step 1) + 16.6 (step 2)	Schwaiger et al., 2004
FIVAR domain	60	400–3200	28	Milles et al., 2017
FimA (A. Oris)	700	400	14	Echelman et al., 2016
FimA (E. Coli)	530 (oxidized) 310 (reduced)	400	42 57	Alonso-Caballero et al., 2018
FimF	420 (oxidized) 270 (reduced)	400	43 55	Alonso-Caballero et al., 2018
FimG	430 oxidized (tu = 1 s) 340 reduced (tu = 0.03 s)	400 (300 pN in clamp)	40 52	Manteca et al., 2017; Alonso-Caballero et al., 2018
FimH lectin domain	Single event: 130 Two events: 100 and 110	400	Single event: 40 Two events: 6 and 36	Alonso-Caballero et al., 2018
FimH pilin domain	360 oxidized 240 reduced	400	38 47	Alonso-Caballero et al., 2018
GB1 domain	180	400	18	Cao et al., 2006; Cao and Li, 2007
GB1 mutant G6-53	Apo: 120 Co^2+^ bound: 150 Co^3+^ bound: 260	400	18	Xia et al., 2019
Gelsolin	Apo: 20 Holo: 40	1000	35	Lv et al., 2014
HγD-crystalin	N-term. domain: 130 C-term. domain: 90	400	30	Garcia-Manyes et al., 2016
I91(formerly I27)::75Gly_5_	200	400	30	Carrion-Vazquez et al., 1999
iLOV domain	100	800	36	Jobst et al., 2015
Leucine-binding protein	70 (intermediate state observed)	1000	120	Kotamarthi et al., 2013b
Maltose-binding protein	75 (intermediate state observed)	400	100	Aggarwal et al., 2011
Protein L	135	400	19	Sadler et al., 2009; Elosegui-Artola et al., 2017
Spectrin domains R13-R18	30	80–800	31	Rief et al., 1999; Randles et al., 2007
Spy0128 E117A (N-ter) (C-ter)	180 250	400	52 52 (with intermediates)	Alegre-Cebollada et al., 2010a
Sumo	125	400	24	Kotamarthi et al., 2013a
Tenascin	∼180	1000	28	Oberhauser et al., 1998
Titin I32 I34 I28 I4 I5	298 281 257 171 155	400	28	Li et al., 2000b, 2002
Titin I91 (formerly I27) (wild type)	200	500	28	Rief et al., 1997; Liu et al., 2018
Titin I91 (formerly I27) mutants	Y9P: 268 V11P: 143 V13P: 132 V15P: 159	600–800	28	Li et al., 2000a
Titin I91 (formerly I27)_(G32C-A75C)	180 oxidized 170–190 reduced	400	12 29	Ainavarapu et al., 2007; Manteca et al., 2017
Titin Z1 Z2	125 174	400	30.8 30.8	Garcia-Manyes et al., 2012
Top7(G90P)	130	400	29	Sharma et al., 2007
Top7(Q3C/T51C)	172 (oxidized) 140 (reduced)	400	13 30	Sharma et al., 2007
Top7	160	400	29	Sharma et al., 2007
Ubiquitin	N-C pulling geometry: 203 Lys48-C pulling geometry: 85	280–310	N-C pulling geometry: 24 Lys48-C pulling geometry: 7.8	Carrion-Vazquez et al., 2003
Xylanase	2–3 unfolding steps, each step: 50	200–6400	89	Stahl et al., 2012; Schoeler et al., 2014

## Pulling Protocols and Cantilever Innovations in Afm-Smfs

The time-dependent evolution of force experienced by the POI in AFM-SMFS experiments can be controlled by applying various pulling protocols (Figure 1F). An early method still commonly in use today is referred to as “constant speed” mode, where the distance between the base of the AFM cantilever and the surface (*z*) is increased at a constant rate. This method only requires open loop positional control of the piezo element in the AFM and is therefore very straightforward to implement, however, open loop operations of piezo elements are generally not recommended due to piezo drift. Other commonly used methods include “force ramp” and “force clamp” modes. In these modes, the photodiode deflection signal is used in a feedback loop to adjust the piezo position such that the POI experiences a tension value set by the experimenter. In force ramp mode, the force is increased linearly with time (Oberhauser et al., 2001; Marszalek et al., 2002). Force clamp can be viewed as a subtype of force ramp with a ramp velocity equal to zero, and the force applied to the POI is held at a constant value (Oberhauser et al., 2001; Popa et al., 2013b). Force ramp and force clamp modes can be used to directly observe force-dependent kinetics of protein unfolding and receptor-ligand complex rupture. Force ramp and force clamp protocols are more prone to external perturbations compared to the constant speed protocol, and the precision of force tuning is limited by many factors, including the response time of the cantilever, drift in the system, and the signal sampling frequencies.

Beyond force ramp, researchers have further developed pulse-chase protocols to study force-induced reactions that can modulate the length of proteins, such as disulfide reduction/oxidation (Liang and Fernández, 2009; Perez-Jimenez et al., 2009; Alegre-Cebollada et al., 2010b, 2014; Kosuri et al., 2012; Kahn et al., 2015; Beedle et al., 2017, 2018; Giganti et al., 2018), domain unfolding (Garcia-Manyes et al., 2007, 2009a,b; Walther et al., 2007; Berkovich et al., 2010; Popa et al., 2013b; Echelman et al., 2016), elastic stretching (Berkovich et al., 2012), and the reversibility of such reactions. In pulse-chase protocols, force clamp is used to apply an initial force pulse to unfold a protein or a series of fingerprints/POI domains. The force pulse triggers a mechanochemical reaction of interest, for example, domain unfolding or disulfide bond cleavage by nucleophiles. The force is then quenched to zero or other sufficiently low value to allow the reverse reaction to take place. The occurrence of the back reaction is then characterized by applying a second force pulse and determining the fraction of event recurrence.

A recently developed pulling protocol, zig-zag force ramp, has enhanced the ability of detecting protein unfolding intermediates (Jacobson et al., 2019; Nash, 2019). The zig-zag force ramp protocol uses open loop piezo control to move the AFM tip away from the surface at a constant speed, followed by reversing direction and moving the tip closer to the surface in a two steps forward/one step backward manner. This up-down cycle is repeated periodically at a low frequency of ∼10 Hz, gradually increasing the distance between the tip and the surface in a stepwise fashion. When combined with precise force measurements and high temporal resolution enabled through the use of custom modified cantilevers (see below), the zig zag protocol was able to detect many intermediate folding states of bacteriorhodopsin not previously observable by conventional constant speed/force ramp measurements (Jacobson et al., 2019).

A related direction of improving measurement techniques in AFM-SMFS is modifying cantilevers for improved time resolution, stability, and force sensitivity (Edwards and Perkins, 2017; Faulk et al., 2017; Sigdel et al., 2018). A simple approach for improving the stability of cantilever-based measurements is to remove the gold coating that is typically found on the backside of silicon-based cantilevers. The gold coating increases reflectivity and increases the photodiode signal, but this comes at a cost of decreased stability and increased thermal noise caused by differential thermal expansion coefficients between the gold and Si layers (Sandberg et al., 2005; Ramos et al., 2007). By removing the gold coating, the bimetallic expansion is eliminated and cantilevers with sub-pN stability can be fabricated (Churnside et al., 2012). While removal of the gold layer improves stability, it may also reveal that instrumental positional drift is a limiting factor, particularly at low frequencies. Follow up work demonstrated that focused ion beam milling of large sections of commercial cantilevers could be used to reduce hydrodynamic drag, improving force precision at low frequencies. Thinning of the remaining ablated support beams on the cantilever further softened the spring constant enabling long term for stability. Furthermore, gold was removed from everywhere on the lever except a small patch at the cantilever head, allowing high reflectivity but minimizing the bimetall effects (Bull et al., 2014). Other shapes including the warhammer (Edwards et al., 2017) and T-shaped cantilevers (Kim and Sahin, 2015) can furthermore improve signals for AFM-SMFS and be combined with imaging modes of AFM. With enhanced SMFS precision at 1-μs, the free energy barrier describing a three amino acid transition could be well reconstructed (Yu et al., 2017). In the future, new modifications and creatively shaped cantilevers can be expected to balance out various performance parameters such as stability, force precision, and time resolution.

## Contour Length Transformations and Elasticity Models

**FIGURE 2:**
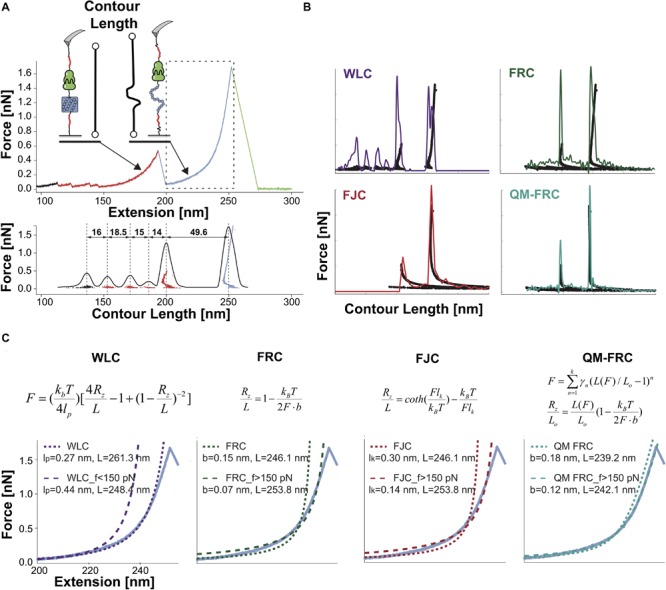
Overview of SMFS data processing by contour length transformation and molecular fingerprinting. **(A)** Top: a typical force vs. extension trace for stretching a multi-domain polyprotein assembled through a mechanically stable receptor-ligand complex. Red shows unfolding and stretching of two low-force marker domains [ddFLN4 (Schwaiger et al., 2004)], followed by unfolding and stretching of a mid-stability marker domain [CBM (Stahl et al., 2012)], followed by rupture of the mechano-stable receptor-ligand complex [SdrG:Fgβ (Milles et al., 2018)]. Bottom: Assembly of a contour length histogram following transformation into contour length space using an elasticity model of choice. Distances between peaks of the contour length histogram are used to make domain assignments to unfolding events in the data trace. **(B)** Four polymer elasticity models were used to transform the data from panel **A**. WLC, worm-like chain; FRC, freely-rotating chain; FJC, freely-jointed chain; QM-FRC, quantum mechanical freely rotating chain. For data traces that span a range of forces from <0.1 nN to >1 nN, the QM-FRC model is preferred. **(C)** Transformation equations of the various non-linear elasticity models and examples of model performance on test data showing stretching of unfolded CBM and rupture of the SdrG:Fgβ complex. The two curves in each plot show two separate fitting regimes below and above 150 pN.

When considering domain unfolding or receptor-ligand unbinding, the escape of the system over the energy barrier is accelerated by force, but it remains stochastic. When measured repeatedly, barrier crossing will be observed to occur over a broad range of positions and forces. This makes it difficult to analyze pulling curves using only force-extension coordinates. The free contour length of a polyprotein, however, is a robust statistical parameter that represents the maximal length of physically possible extension in a given folding state. The contour length of the system will theoretically be the same for a given folding state, regardless of the force in the system at any given time. As such it is a robust means to visualize and analyze SMFS data (Figure 2A), and can be used to identify unfolding events for a POI. The additional contour length that is added to the tethered polyprotein following domain unfolding can be estimated simply by the length of the polypeptide released from the protein secondary/tertiary structures during protein unfolding. By knowing the amino acid sequence length of a domain, as well as its folded end-to-end length, we can generate expected values for the change in contour length that should be observed when a given domain unfolds. This is given by the equation ΔL_c_ = (0.365 nm/AA) × (# AAs in POI) − L_f_, where ΔL_c_ is the expected contour length increment, 0.365 nm is the approximate contour length per amino acid of a protein, and L_f_ is the folded end-to-end length of the domain (typically <5 nm) (Dietz and Rief, 2006; Puchner et al., 2008b; Puchner and Gaub, 2009). One source of error in contour length transformations is pulling on molecules that are not positioned directly below the cantilever tip. The distances of these off-axis molecules represent the projection of the true molecular extension onto the vertical axis, tending to shorten the observed contour length increments. To address this, feedback systems have been developed to center molecules directly under the tip (Walder et al., 2018a).

Since receptor-ligand rupture typically results in loss of the tether between the cantilever and the surface, calculation of ΔL_c_ upon rupture does not have the same physical meaning for receptor-ligand rupture experiments as for domain unfolding experiments, however, ΔL_c_ calculations can be incorporated for fingerprinting of receptor-ligand interaction curves as well. Also, tethered protein receptor-ligand (Bertz et al., 2009; Kim et al., 2010; Pernigo et al., 2010; Berkemeier et al., 2011; Vera and Carrión-Vázquez, 2016; Milles and Gaub, 2019) and DNA systems (Halvorsen et al., 2011; Yang et al., 2016) have been reported where the rupture of a molecular interaction results in extension of a flexible tether providing a known contour length increment. Therefore, ΔL_c_ analysis can be highly applicable not only to domain unfolding studies but also to receptor-ligand rupture experiments.

To calculate contour length increments (ΔL_c_), polymer elasticity models such as the worm-like chain (WLC) (Bustamante et al., 1994), the freely jointed chain (FJC) (Ortiz and Hadziioannou, 1999), the freely rotating chain (FRC) (Livadaru et al., 2003), or quantum mechanical FRC (QM-FRC) (Hugel et al., 2005) models are applied to transform the force-extension curve using a one-to-one mapping into force-contour length space. A widely used model is an interpolation formula of the WLC (Bustamante et al., 1994), and is appropriate for ideal stiff chains. This model mathematically describes the stretching of unfolded proteins, DNA, RNA, and other biopolymers reasonably well up to forces around 150 pN. To extend the theoretical treatment to higher force regimes, Livadaru et al. (2003) proposed an FRC model for semiflexible polymer chains made up of discrete segments. For the same purpose, quantum mechanical corrections based on the WLC model were proposed to account for polypeptide backbone stretching in the high-force range of up to two nanonewtons (Hugel et al., 2005). A combination of the WLC model in low force regime and FRC model in the high force regime with quantum mechanical correction (QM-FRC) can be used to analyze AMF-SMFS data that spans a wide force range from tens of piconewtons up to two nanonewtons (Figures 2B,C).

Depending on the solvent environment, the effects of monomer side chains may become evident in the elastic response of individual biopolymers. A recent study by Cai et al. (2019) showed that a more consistent fitting could be achieved using a new TSQM model that upgrades the previous modeling work with structure-relevant terms. Given the importance of elastic stretching behavior in AFM-SMFS, isomerization reactions within monomer units of mixed synthetic/protein polymer systems can also become problematic, and blur contour length histograms. To address this, intrinsically disordered elastin-like polypeptides have been incorporated as linkers, avoiding the *trans*-gauche isomerization of PEG-linkers that occurs around 300 pN (Oesterhelt et al., 1999; Liese et al., 2017; Ott et al., 2017).

## Theoretical Models of the Energy Landscape

The conceptual free energy landscape is a high dimensional surface upon which proteins sample many conformations on their way to the folded state. Due to the importance of protein folding, misfolding, and conformational-sampling in biological systems, quantifying energy landscapes is highly informative for the understanding of molecular behavior and can inform the development of new therapies. Using AFM-SMFS, we can perturb the energy landscape and measure the influence of force on transition rates from one state to another. This allows us to characterize and depict the energy landscape (Hummer and Szabo, 2001; Woodside and Block, 2014) using appropriate theoretical models to describe the transition of the system over an energy barrier under the influence of an external force.

Three models used regularly to describe this problem are the Bell-Evans (Bell, 1978; Evans and Ritchie, 1997), Dudko-Hummer-Szabo (Dudko et al., 2006, 2008; Dudko, 2016) and Friddle models (Friddle et al., 2012; Noy and Friddle, 2013). The Bell-Evans model predicts a linear dependence of the rupture force on the natural logarithm of the loading rate, and gives access to the intrinsic off rate *k*_off_ and the position of the energy barrier Δx. This framework was further developed by Dudko et al. (2008) by specifying the shape of the free-energy surface, and accounting for changes in Δx as the force rises. In addition to *k*_off_ and Δx, the Dudko model further provides the height of the activation energy barrier (ΔG^‡^). Friddle et al. (2012) developed a framework to account for rebinding in a low force equilibrium regime. Further theoretical treatments of this problem have been developed to reconstruct the entire one dimensional free-energy landscape from SMFS data (Rhee and Pande, 2005; Woodside and Block, 2014). By deconvoluting instrument effects (Walder et al., 2018b), such reconstruction approaches have been validated on DNA hairpins (Gupta et al., 2011) and proteins (Yu et al., 2012) and found agreement between various single-molecule manipulation techniques (Woodside and Block, 2014; Manuel et al., 2015). A full coverage of theoretical work covering this problem is, however, beyond the scope of this work.

## Surface Chemistry

**FIGURE 3:**
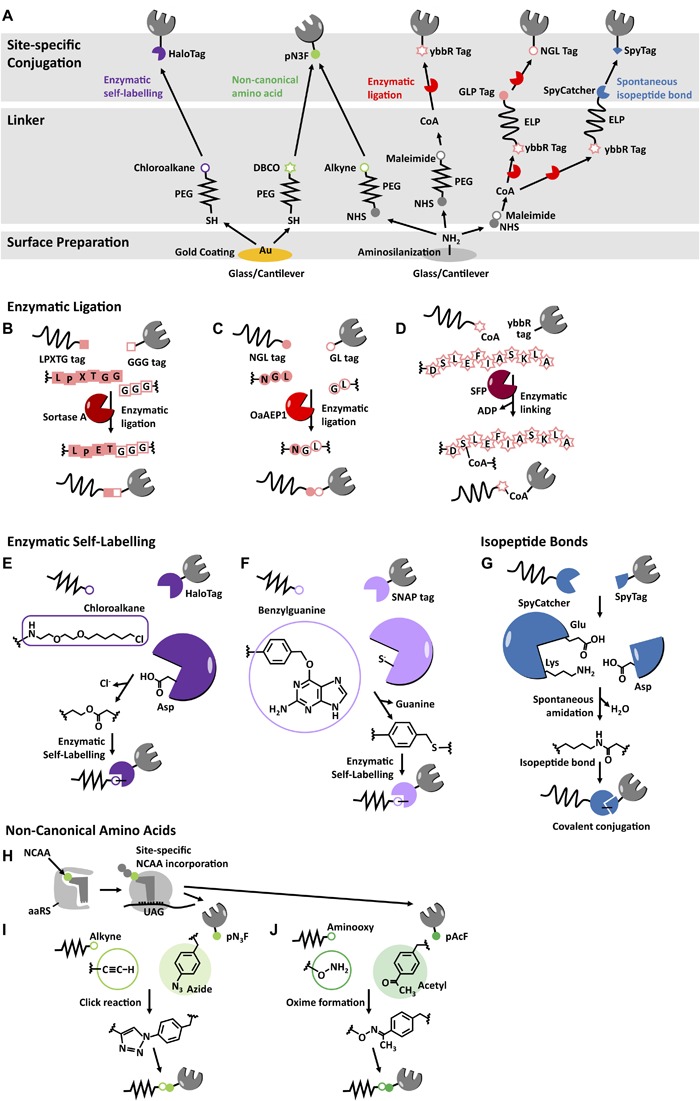
Surface chemistry, linkers, and site-specific immobilization methods for SMFS. **(A)** Overview of cantilever and glass preparation for AFM-SMFS. Chemical functionalization of the substrate surface by gold-coating or aminosilanization is followed by passivation and attachment of a suitable flexible linker (typically PEG or ELP) containing a functional end group. Target molecules can be further immobilized site-specifically by several strategies: Enzymatic ligation using **(B)** LPXTG tag/GGG tag/Sortase A, **(C)** NGL tag/GL tag/OaAEP1, and **(D)** ybbR tag/CoA/SFP; Enzymatic self-labeling using **(E)** HaloTag with chloroalkane derivatives or **(F)** SNAP tag with benzyl group of benzylguanine; Spontaneous isopeptide bonds formation using **(G)** SpyTag/SpyCatcher, SnoopTag/Snoop catcher, and isopeptag/Pilin-C systems; Non-canonical amino acids incorporated by **(H)** amber suppression with **(I)** p-azidophenylalanine (pN_3_F) for click reactions with alkyne or DBCO compounds or **(J)** p-acetylphenylalanine (pAcF) for oxide formation with an aminooxy group.

Although non-specific adsorption of polyproteins can work well for measuring protein unfolding, generally when receptor-ligand interactions are the objects of study, covalent attachment chemistry is desired. This avoids the possibility of receptors on the cantilever becoming clogged or blocked by ligand molecules that were picked up from the surface. Surface chemistry for AFM-SMFS can be done differently with a wide range of strategies depending on the design of fingerprint domains and linkers (Figure 3A). One key distinction is between methods that allow for site-specific attachment at a known residue in the protein and those that result in a statistical distribution of anchor points within the molecule (e.g., through lysine residues). Whatever surface chemistry and linkers are used, experiments should be designed in such a way so as to maximize data quality and quantity, not hinder specific protein interactions, and not create stretching or folding artifacts in the data analysis.

Chemical functionalization of cantilevers and substrate surfaces is usually required for further immobilization of target proteins. One way to prepare the substrate surface is using gold. Gold is a very stable and inert material and reacts readily with the thiol group on cysteine, forming a gold-sulfur bond so that thiol-containing molecules can be directly immobilized on gold surfaces. Gold-coated substrates and cantilevers are commercially available and also easily prepared. Due to the ease and convenience of this method, many AFM-SMFS measurements especially in the early years were performed using cysteine thiol-gold chemistry, and the technique remains in use today.

Another way to prepare the substrate surface is silanization. Silicon or silicon nitride cantilevers and glass have silanol groups on their surfaces, and these silanol groups can be functionalized with organic silanes carrying amine or carboxyl groups (Becke et al., 2018). Aminosilanization has been widely applied and standardized for AFM-SMFS (Zimmermann et al., 2010). Further immobilization steps can be performed by reacting amino groups with an N-hydroxysuccinimide (NHS) group. In many of the biological immobilization protocols, aminosilane is the starting layer for further derivatization.

## Elastic Linkers

Proper flexible linkers are necessary for passivation of the surface to achieve very low non-specific interactions and for providing proper binding orientation with low steric hindrance away from the surface. The most common linkers are PEG (polyethylene glycol) polymers. PEGs are linear, highly flexible with well-characterized elastic behavior, and also commercially available with a wide range of functional groups at the ends including NHS, maleimide and azide groups. PEGs provide well-passivated surfaces and provide functional groups for further derivatization. Some disadvantages of PEG include possible polydispersity and a *trans*-gauche to all-*trans* isomerization reaction that sets in around 300 pN of tension. This isomerization can distort contour length analysis for systems at high force (Oesterhelt et al., 1999; Liese et al., 2017).

More recently, elastin-like polypeptides (ELP) have been developed as linkers (Ott et al., 2017). ELPs are composed of a repetitive GXGVP motif, where X can be any amino acid except proline. They are intrinsically disordered and provide added contour length and high flexibility, which are suitable for surface passivation. Also, since ELPs are encoded at the genetic level and expressed in bacteria, they are completely monodisperse with atomically defined lengths and compositions. These features make the use of ELPs a highly accurate measurement technique for analysis of contour length increments (Ott et al., 2017). Site-specific and orthogonal functional groups/peptide tags as well as fusion fingerprint domains can be introduced at the DNA level for further immobilization (Figure 3A).

## Site-Specific Immobilization Tags

Site-specific immobilization allows precise control over the geometrical loading configuration with dramatic effects on the observed mechanical response of protein domains and receptor-ligand complexes. Depending on the biological system being studied, it may be important to study the native pulling geometry experienced by the protein *in vivo.* For synthetic systems, the pulling geometry can be varied to optimize measurement performance or reveal insights into internal stiffness axes within the molecule (Dietz et al., 2006). Site-specific methods can furthermore provide higher yields of useable force-extension curves than non-specific or random covalent immobilization procedures (Walder et al., 2017). Site-specific conjugation can also reduce non-specific interactions since contaminating proteins in the sample are not linked to the surface during the conjugation reaction. This can provide higher accuracy, higher yield and generally more reliable results.

A simple site-specific method that is widely used is through cysteine. Cysteines are somewhat rare in proteins and spontaneously react with gold and maleimide. Genetically encoded point cysteine mutations can be used to conjugate a target protein to a maleimide-terminated PEGylated surface or cantilever. However, this method is limited partly due to hydrolysis of maleimides. Recently, several other methods were developed, and below we illustrate several strategies for site-specific immobilization of target molecules for AFM-SMFS (Figure 3; Banerjee and Howarth, 2018; Wang and Wu, 2018).

### LPXTG Tag/GGG Tag/Sortase A

Sortase A from *S. aureus* recognizes an LPXTG tag at the C-terminus of a target protein, cleaves the bond between threonine and glycine, and ligates the target to a second protein containing an N-terminal oligo G motif (Figure 3B; Theile et al., 2013). One additional amino acid is required at the end of the LPXTG tag for proper binding of Sortase A. Depending on its accessibility, the N-terminal oligo G motif can contain between one and five glycines, however, three glycines (GGG tag) are generally sufficient. The Sortase system exhibits a high micromolar K_m_ value, requiring high concentrations of the substrates. This system has been used for AFM-SMFS for immobilization of protein directly from cell lysate (Srinivasan et al., 2017) or in systems where an LPETGG tag and GGG tag have been used to assemble polyproteins posttranslationally or to attach high-strength Dockerin handles to proteins (Durner et al., 2017; Garg et al., 2018; Liu et al., 2018).

### NGL Tag/GL Tag/OaAEP1

Asparaginyl endopeptidase isolated from the plant *Oldenlandia affinis* (OaAEP1) recognizes an NGL tag at the C-terminus of the target protein, cleaves the bond between asparagine and glycine, and ligates it to an N-terminal GL tag (Figure 3C; Harris et al., 2015). Recently engineered OaAEP1 shows fast, apparently irreversible and highly efficient ligation at neutral pH at RT (Yang et al., 2017). The OaAEP1 system has several advantages compared to sortase. It shows faster and irreversible ligation and does not require any metal ions, while Sortase A shows slow reaction, and requires Ca^2+^ and a longer peptide tag. However, preparation of OaAEP1 requires the additional step for activation under acidic conditions. OaAEP1 has been used for protein immobilization onto surface-based binding assays and also used to posttranslationally assemble polyproteins for AFM-SMFS (Ott et al., 2018; Deng et al., 2019).

### ybbR Tag/CoA/SFP

The 11 amino acid ybbR tag (DSLEFIASKLA) is recognized by 4′-phosphopantetheinyl transferase (SFP) and covalently linked through serine to coenzyme A (CoA) (Figure 3D; Yin et al., 2005). While peptide tags for Sortase A and OaAEP1 should be at the termini, the ybbR tag is more flexible because it can be located at any accessible position in the protein. The ybbR tag/SFP system is widely used as a standard immobilization method for AFM-SMFS with a combination of aminosilanization (Zimmermann et al., 2010; Jobst et al., 2013; Baumann et al., 2017; Ott et al., 2017). Amino groups react to NHS group from hetero-bifunctional PEG (NHS-PEG-Maleimide) or from sulfosuccinimidyl 4-(N-maleimidomethyl)cyclohexane-1-carboxylate (sulfo-SMCC). Then, the thiol group from CoA reacts with maleimide forming a monolayer of CoA. Finally the POI carrying a ybbR tag is site-specifically anchored to the surface using SFP-mediated ligation to CoA.

### HaloTag

Haloalkane dehydrogenase (HaloTag) is a bacterial enzyme of ∼33 kDa that spontaneously forms a covalent ester bond with chloroalkane derivatives (Figure 3E). By modifying surfaces with chloroalkane-derivatized PEGs, and producing the POI as a HaloTag fusion, site-specific immobilization of proteins for AFM-SMFS studies can be readily achieved (Taniguchi and Kawakami, 2010; Popa et al., 2013a).

### hAGT/SNAP Tag

The hAGT or “SNAP” tag (Keppler et al., 2003) binds covalently to the benzyl group of benzylguanine, releasing guanine (Figure 3F). PEGs or thiols carrying the benzylguanine group can be immobilized onto surfaces based on self-assembled thiol monolayers on gold or using silane chemistry on glass surfaces or silicon cantilevers. The gene encoding the POI is fused with DNA sequence encoding the SNAP tag. Expressing this construct results in a 19 kDa SNAP fusion domain attached to the POI. This approach has been demonstrated as a useful site-specific immobilization method for single-molecule force spectroscopy (Kufer et al., 2005; Fichtner et al., 2014).

### Isopeptide Bonds

Isopeptide bonds are intramolecular covalent amide bonds formed outside of protein backbone between amino acid side chains. Isopeptide bonds form spontaneously upon nucleophilic attack of a primary amine from a lysine side chain toward a carboxamide/carbonyl group of asparagine/aspartic acid in close proximity to a catalytic glutamic acid (Kang et al., 2007). Proteins having isopeptide bonds have been engineered by dissecting the fold into two fragments and utilizing spontaneous covalent isopeptide bond formation upon fold reconstitution to site-specifically link targets together (Zakeri and Howarth, 2010; Zakeri et al., 2012; Veggiani et al., 2016). Isopeptide bond formation is fast, efficient, irreversible, and robust to diverse conditions (Zakeri et al., 2012), and is being increasingly used for site-specific immobilization of proteins for AFM-SMFS. The Spytag/Spycatcher system is perhaps the most well known isopeptide bond system, comprising the second immunoglobulin-like collagen adhesin domain of *S. pyogenes* which is stabilized by spontaneous isopeptide formation between Lys and Asp. This fold was rationally engineered and split into two parts: 13 amino acid SpyTag and the remainder of the domain, SpyCatcher (Figure 3G; Zakeri et al., 2012). SpyTag can be inserted at the protein terminus or internally in the sequence and remains reactive as long as it is accessible and can form the structure with SpyCatcher. SpyCatcher part can be further divided into two parts: KTag/SpyLigase or BDTag/SpyStapler for peptide-peptide ligation (Fierer and Veggiani, 2014; Wu et al., 2018). This SpyTag/SpyCatcher system was recently used for immobilization of a cellulose binding module onto a cantilever for AFM-SMFS (Griffo et al., 2019).

The SnoopTag/Snoop catcher system was derived from a C-terminal domain of adhesin RrgA from *S. pneumonia*, which is stabilized by spontaneous isopeptide between Lys and Asn and engineered into two parts: 12 amino acid SnoopTag and SnoopCatcher (Veggiani et al., 2016). This adhesin RrgA domain was also divided and engineered into three parts: SnoopTagJr/DogTag/SnoopLigase for peptide-peptide ligation (Buldun et al., 2018). Owing to fully orthogonal reactivity of SnoopTag/SnoopCatcher pair and SpyTag/SpyCatcher pair, they can be used at the same time with no cross-reactivity (Veggiani et al., 2016). The isopeptag/Pilin-C system was derived from the major pilin protein Spy0128 from *S. pyogenes* and is stabilized by spontaneous isopeptide bond formation between Lys and Asn. The domain was engineered at the C-domain into two parts: 16 amino acid Isopeptag and pilin-C (Zakeri and Howarth, 2010). This protein was also engineered differently by splitting at the N domain producing isopeptag-N and pilin-N (Veggiani et al., 2014).

### Non-canonical Amino Acids

Non-canonical amino acid (NCAA) incorporation is a sophisticated strategy to introduce new functional groups into proteins (Kim et al., 2013). Natural amino acids cover only a very limited range of functional groups and because the same functional groups are repeatedly incorporated into multiple sites in typical protein, their chemical selectivity is poor. These limitations can be overcome by introducing unique bio-orthogonal functional groups into target proteins via site-specific NCAA incorporation. To date, a variety of unique amino acids and their orthogonal aminoacyl-tRNA synthetase (aaRS) pairs have been developed (Wang et al., 2006). The target amino acid with a unique functional group is recognized by a corresponding aaRS and takes part in the translational machinery at the site of a corresponding codon (typically the amber codon) (Figure 3H). Depending on the choice of the NCAA, site-specific immobilization for AFM-SMFS can be highly specific, bioorthogonal, and efficient. For example, click chemistry with an azide group is often used. NCAAs having azide groups such as p-azidophenylalanine (pN_3_F) are incorporated into target proteins at a desired site, and this target protein can be easily immobilized onto alkyne- or DBCO-terminated PEGylated surfaces (Figure 3I; Deiters et al., 2004; Maity et al., 2016; Yu et al., 2019). Also, p-acetylphenylalanine (pAcF) can be introduced for immobilization to aminooxy-terminated PEGylated surface by oxime formation (Figure 3J; Cho et al., 2011; Hallam et al., 2015). While many of the other methods described require longer peptide tags or require the ligation site to be located at the terminus of the protein, NCAA incorporation changes only a single amino acid and therefore minimally perturbes the target protein. Also NCAA-based attachment is not restricted to the protein terminus but can be achieved in the middle of the amino acid sequence. As such, this method provides high flexibility in terms of selection of pulling positions for AFM-SMFS. The downside to NCAA incorporation is that due to poor efficiency of NCAA incorporation at the ribosome, the yield of functional protein obtained during an expression/purification run is typically much lower than that achieved with the wild type sequence. This limitation is perhaps not so severe for studies focusing only on single-molecule approaches, however, if bulk biochemical assays (e.g., calorimetry, ELISA, thermal denaturation analysis, etc.) are to be performed in addition to single-molecule measurements, then the limited amount of material obtained from NCAA incorporation may be problematic.

## Conclusion

AFM-SMFS is a well established technique in the nanobio sciences that is ideally suited for studying molecular mechanical properties. Although molecular mechanical properties are highly important in biology, a majority of cell and molecular biologists do not think of their systems in mechanical terms and therefore our understanding of the influence of forces on protein and cells remains in its infancy. One reason for this is that force as an experimental parameter is difficult to control. Here we attempted to outline the various measurement configurations for AFM-SMFS, as well as relevant theory and algorithms for high-throughput curve selection/analysis. Finally, we summarized state-of-the-art methods for anchoring molecules to surfaces using site-specific bioconjugation methods for AFM-SMFS. Using these next-generation improved methods for SMFS, we hope to assist the community in their endeavor to improve data quality, yield, and reproducibility in a concerted effort to enhance our understanding of molecular biomechanical systems.

## Author Contributions

All authors listed have made a substantial, direct and intellectual contribution to the work, and approved it for publication.

## Conflict of Interest

The authors declare that the research was conducted in the absence of any commercial or financial relationships that could be construed as a potential conflict of interest.
